# Poly‐l‐Lactic Acid Is Effective and Safe to Treat the Perioral Area: Report of Successful Cases

**DOI:** 10.1111/jocd.16738

**Published:** 2024-12-19

**Authors:** Cristiane Cesar Evangelista, Mayra Ianhez, Gisele Viana de Oliveira, Rosemarie Mazzuco

**Affiliations:** ^1^ CCE Private Practice Belo Horizonte Brazil; ^2^ Federal University of Goiás Goiania Brazil; ^3^ Faculty of Medical Sciences of MG Brazil and Santa Casa de Misericórdia de BH Belo Horizonte/MG Brazil; ^4^ RM Private Practice Carazinho/RS Brazil

**Keywords:** facial rejuvenation, perioral wrinkles, poly‐l‐lactic acid

Dear Editor,

Perioral rhytids are among the earliest signs of facial aging [1, 2], particularly challenging to treat, and they are associated with sagginess and lipoatrophy on an anatomical area of great mobility [1].

Poly‐L‐Lactic Acid (PLLA‐SCA) is an absorbable biostimulator that activates immune cells, leading to increased collagen production [3, 4]. Although most side‐effects are mild and transient [3, 4], the risk of nodules or long‐term granulomas are major concerns when PLLA is used in areas of thin skin or high mobility, such as perioral area [5]. Studies that reported the risk of nodules in these areas utilized a dilution of 4‐5 mL of sterile water per vial [5], a technique no longer in practice.

The authors have successfully used PLLA‐SCA to treat the perioral area in recent years; over the past 12 months, we evaluated 12 healthy female patients who underwent this treatment. None were smokers or had autoimmune diseases, and all were instructed to avoid anti‐inflammatories or corticosteroids during treatment. The study adhered to the Helsinki's Declaration, and patients provided informed consent.

Each PLLA‐SCA (Sculptra; Dermik Laboratories, NJ) vial was reconstituted immediately before the procedure using 14 mL of sterile water and 2 mL of lidocaine, resulting in a 16 mL final dilution, commonly used for non‐facial applications.

Antisepsis was performed (2% chlorhexidine soap), followed by topical anesthesia (4% lidocaine) and infraorbital nerve block. Injections were administered with a 22Gx50mm cannula using the “fanning technique.” [6] PLLA‐SCA was distributed employing 1 mL per side in the nasolabial fold (9 mg/side) and 2 mL (9 mg/side) in the upper lip, excluding the philtrum; on three monthly sessions.

Standardized photographs were taken before and 3 months after the last session, capturing anterior and lateral views at rest and during muscle contraction of the mouth. After 12 months, patients completed a satisfaction questionnaire, including an assessment of papules or nodules in the treated area. Three dermatologists who did not participate on the injections rated before and after wrinkles scores, using the Wrinkle Severity Scale (WSS), the philtrum length (from columella to lip) and the Global Aesthetic Improvement Scale (GAIS).

Patients ages varied from 39 to 72 years (mean 53.1). The average ratings from the evaluators are shown in Table 1. Regarding the WSS, 10 patients experienced wrinkle improvement (at rest and during contraction) (Figure 1). GAIS assessments revealed good to very good improvement scores on five patients (41%) (Table 1). One interesting finding was the reduction in the philtrum length observed in five patients. No volumization or motor dysfunction was observed, although subtle eversion of the upper lip led to high satisfaction in some patients. All patients referred minimal discomfort during injections, with transient edema lasting up to 24 h. No papules, nodules, or persistent side‐effects were reported, and all patients expressed overall satisfaction with the results.

**TABLE 1:** Average of the scores of the three independent evaluators.

Patient	Age	WSS^a^ before (at rest)	WSS^a^ after (at rest)	WSS^a^ before (contracted)	WSS^a^ after (contracted)	GAIS^b^ (at rest)	GAIS^b^ (contracted)	Philtrum's decrease^c^
1	59	3.66	3.66	4	4	3	3	1.66
2	41	2	2	4	3	3.66	4.33	2.33
3	58	2	1	2.33	1.33	5	4.66	3
4	63	3	2	3.33	3.33	4.33	3	2.33
5	72	3	2	4	4	3.66	3	2
6	65	2.66	2	4	3.33	4	4.33	2.66
7	55	2	1.66	2	2	4	3.33	3
8	49	1.66	1.33	2.66	2	3.33	3.33	1.66
9	39	2	1.66	3	2	3.66	4	3
10	50	1.66	1.33	3	3	2.66	2.66	2.33
11	39	2	2	3.33	2.33	3.33	3.66	2.33
12	48	2	1	2	1.66	4	4	2.66
Average	53.1 (39–72)	2.30	1.80	3.13	2.66	3.72	3.60	2.41

^a^ WSS, Wrinkle Severity Scale. 1—No wrinkles; 2—Fine lines; 3—Moderate wrinkles; 4—Deep wrinkles.

^b^ GAIS, Global Aesthetic Improvement Scale. 1—Worsening; 2—No improvement; 3—Slight improvement; 4—Good improvement; 5—Very good improvement.

^c^ Philtrum's decrease (lip–columella length). 1—There was an increase in distance. 2—No change. 3—There was a decrease in distance. PS: We consider an average higher than 2.66 as positively decreasing on philtrum.

**FIGURE 1:**
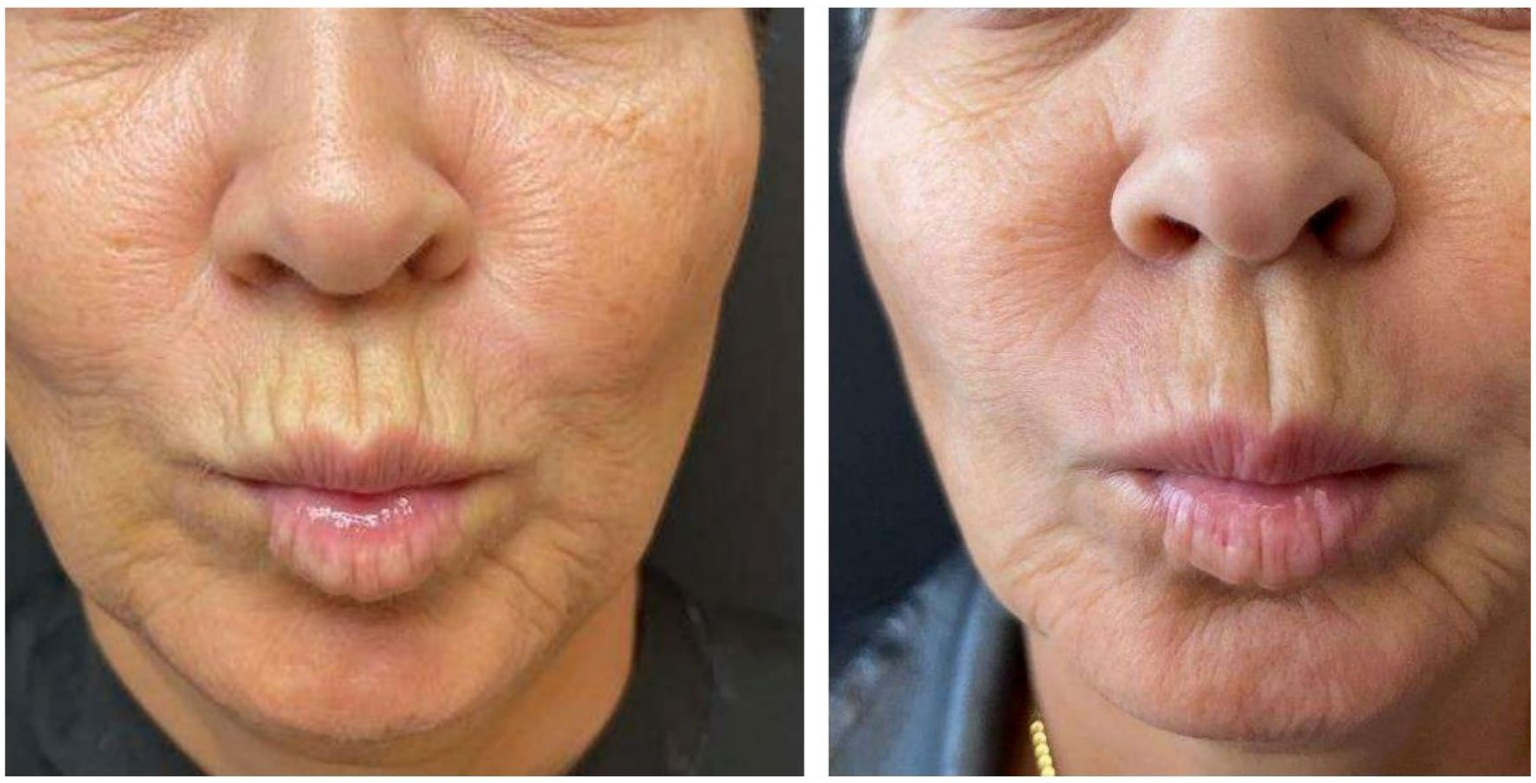
Before and 3 months after the last session of treatment of a 55 years old female. Observe the enhancement of perioral wrinkles and skin quality, without any modifications to the volume or muscular action.

The perioral region is highly complex, requiring a combination of procedures to achieve optimal aesthetic result [2]. Some patients require a global approach to target skin quality, wrinkles and skin flaccidity. PLLA‐SCA has been proven to be safe and effective to improve volume, skin quality, and laxity [3, 4, 5]. With advancements in dilution and technique, our group has observed a significantly lower incidence of nodules compared with the previous dilution of PLLA‐SCA [5], making it suitable for the perioral region. We opted for the dilution of 16 mL, widely used for off‐face areas, including neck, where the skin is similarly thin and subject to muscular contraction, as in the perioral area. In our study, in addition to wrinkle improvement, a reduced philtrum length and a slight lip eversion were observed, likely due to collagen‐induced tissue retraction [7]. The absence of nodules can be explained to the low dose injected. In conclusion, our findings suggest that PLLA‐SCA may be safe and effective to treat the nasolabial fold and upper lip area.

## Conflicts of Interest

The authors declare no conflicts of interest.

## Data Availability

The data supporting the findings of this study are available upon reasonable request from the corresponding author.
